# Secondary transfer of emergency stroke patients eligible for mechanical thrombectomy by air in rural England: economic evaluation and considerations

**DOI:** 10.1136/emermed-2019-209039

**Published:** 2020-11-10

**Authors:** Diarmuid Coughlan, Peter McMeekin, Darren Flynn, Gary A Ford, Hannah Lumley, David Burgess, Joyce Balami, Andrew Mawson, Dawn Craig, Stephen Rice, Phil White

**Affiliations:** 1 Population Health Sciences Institute, Newcastle University, Newcastle upon Tyne, UK; 2 Faculty of Health and Life Sciences, Northumbria University, Newcastle upon Tyne, UK; 3 School of Health and Social Care, Teesside University, Middlesbrough, UK; 4 Oxford University Hospitals NHS Trust, Oxford, UK; 5 Institute of Neuroscience (Stroke Research Group), Newcastle University, Newcastle upon Tyne, UK; 6 North East and North Cumbria Stroke Patient & Carer Panel, Newcastle upon Tyne, UK; 7 Kellogg College, University of Oxford, Oxford, UK; 8 Great North Air Ambulance, Northumberland Wing, The Imperial Centre, Darlington, UK

**Keywords:** stroke, thromboembolic diseasex, management, emergency ambulance systems, helicopter retrieval, emergency care systems, remote and rural medicine

## Abstract

**Background:**

Mechanical thrombectomy (MT) is a time-sensitive emergency procedure for patients who had ischaemic stroke leading to improved health outcomes. Health systems need to ensure that MT is delivered to as many patients as quickly as possible. Using decision modelling, we aimed to evaluate the cost-effectiveness of secondary transfer by helicopter emergency medical services (HEMS) compared with ground emergency medical services (GEMS) of rural patients eligible for MT in England.

**Methods:**

The model consisted of (1) a short-run decision tree with two branches, representing secondary transfer transportation strategies and (2) a long-run Markov model for a theoretical population of rural patients with a confirmed ischaemic stroke. Strategies were compared by lifetime costs: quality-adjusted life years (QALYs), incremental cost per QALY gained and net monetary benefit. Sensitivity and scenario analyses explored uncertainty around parameter values.

**Results:**

We used the base case of early-presenting (<6 hours to arterial puncture) patient aged 75 years who had stroke to compare HEMS and GEMS. This produced an incremental cost-effectiveness ratio (ICER) of £28 027 when a 60 min reduction in travel time was assumed. Scenario analyses showed the importance of the reduction in travel time and futile transfers in lowering ICERs. For late presenting (>6 hours to arterial puncture), ground transportation is the dominant strategy.

**Conclusion:**

Our model indicates that using HEMS to transfer patients who had stroke eligible for MT from remote hospitals in England may be cost-effective when: travel time is reduced by at least 60 min compared with GEMS, and a £30 000/QALY threshold is used for decision-making. However, several other logistic considerations may impact on the use of air transportation.

**Key messages**

**What is already known on this subject**

- Mechanical thrombectomy is a proven highly cost-effective procedure.
- Air transport has been used successfully for rural patients who had stroke eligible for mechanical thrombectomy in various jurisdictions.

**What this study adds**

- This modelling study indicated that secondary transfer of early-presenting rural patients who had stroke eligible for mechanical thrombectomy by air is an economically viable strategy if the time saved was 60 min and a £30 000/QALY threshold is used for decision-making.
- For late presenters, the value argument is less contentious as air transport is not cost-effective.

## Introduction

Mechanical thrombectomy (MT) is an endovascular procedure that significantly improves functional outcomes for patients who had ischaemic stroke caused by large artery occlusion by physically removing clot from the blocked artery in the brain.^1^ Delivery of MT involves a complex care intervention and pathway including specialist angiographic suites, a trained and experienced acute stroke and neuroendovascular teams with anaesthetic, critical care and preferably neurosurgical support. Recent research suggests that around 10% of patients who had stroke admitted to hospital in the UK would benefit from MT.^2^ It is a highly cost-effective treatment when used either alone or in conjunction with intravenous thrombolysis.^3^ The effectiveness of both treatments is extremely time-dependent, with efficient emergency transportation deemed a critically important factor for achieving better long-term outcomes for patients.^4^ Currently, only urban-based neuroscience centres in England can deliver MT services, creating potential inequity of provision for rural patients—especially in areas with low population density that preclude a local thrombectomy service ever being economically viable.

To improve equity of access for eligible rural patients served by smaller hospitals, the use of aeronautical transportation for secondary (interhospital) transfer has been proposed.^5^ Empirical research comparing the use of Air Ambulance (incorporating helicopter emergency medical services (HEMS)) with ground emergency medical services (GEMS) for secondary transfer of patients who had stroke has identified a variety of logistical, geographical and regulatory factors.^6–8^ The current international debate regarding the optimal paradigm for acute (emergency) stroke services includes HEMS transportation.^9^ From an NHS perspective, there is currently a need for the majority of regions in England to undertake secondary transfer by GEMS (ie, neuroscience centres take eligible patients with MT after initial assessment in a hyperacute stroke unit known as the *drip-and-ship* paradigm).

Given the under provision of neurointerventional teams and the geographically uneven distribution of neuroscience centres, NHS commissioners are beholden to examine service delivery options for providing MT, particularly those from remote locations.^10^ For many health systems such as the NHS, any newly proposed service delivery is accompanied by evidence of cost-effectiveness. This study aimed to evaluate whether secondary transfer (ie, drip-and-ship) by HEMS offers a cost-effective delivery option compared with GEMS using a decision analytic model for eligible rural patients who had stroke in England based on receiving optimal advanced imaging and treatment, prior to secondary transfer.

## Methods

### Relevant NHS population

An ‘unavoidably small and remote’ hospital is defined by the NHS in England as a hospital serving a population of ≤2 00 000 people who are domiciled more than 60 min travel by road from the nearest (major acute) hospital.^11^ We chose these hospitals, as they will never become MT centres—given that 1000–1200 ischaemic stroke admissions per annum (or a catchment population of ~1 000 000) is the likely minimum throughput required to sustain a stand-alone 24/7 MT centre’s experience and performance in England.^12^ Entry into our model is based on patients already having a confirmed large artery occlusion ischaemic stroke after brain CT and (arch through brain) CT angiography examinations, treated with intravenous thrombolysis (where indicated) and deemed eligible for secondary transfer for MT.

The average age in the UK for someone to have a stroke is 72 years for men and 78 years for women, according to Sentinel Stroke National Audit Programme (SSNAP) data.^13^ To demarcate MT-eligible patients based on time, we stratify into early and late presenters. In our base-case analysis, we define early presenters as patients receiving an arterial puncture within 360 min from the time of onset of stroke. This cut-off point was identified from a meta-analysis of five randomised trials.^14 15^ We defined late presenters as those who had arterial puncture after 360 min. Working backwards from puncture time there is a need to account for inevitable travel time from initial emergency call to reach remote hospital (assumed ≥150 min) and subsequent door-in to door-out at remote hospital (assumed ~60 min) and door-to-puncture time at MT centre (assumed ~30 min). Therefore, we focus simulation modelling on puncture time between 240 and 360 min.

### Model structure and data sources

The economic model comprised a decision analytical model with two components: (1) a ‘short-run’ decision tree with two branches, representing secondary transfer transportation strategy (HEMS vs GEMS) and (2) a long-run Markov model with 3-month cycle length. The decision tree modelled the acute phase (up to 3 months) and the Markov model captured the lifetime health outcomes, incorporating the probability of a stroke reoccurrence. Health outcome states in stroke research typically use the modified Rankin Scale (mRS)^16^ that measures the degree of in/dependence in daily activities after a stroke. Thus, patients who had stroke with mRS levels 0, 1 or 2 at 90-day poststroke are considered to be functionally independent (no symptoms or slight disability). Those with mRS level 3, 4 or 5 are considered dependent and have moderate to severe disability (mRS 6=deceased). On entering the Markov model, the health outcome probability of a patient was based on trial evidence that a relatively linear relationship exists between time to reperfusion and 90-day mRS outcome.^15^ Transition probabilities after 1 year from one health state (mRS) to another and 90-day mRS outcome probabilities from intravenous thrombolysis treatment are taken from a previous economic evaluation.^17^

To estimate the effect of a reduction in travel time, the results of an academic collaboration of seven randomised controlled trials was used for early presenters,^14 15^ whereas for late presenters, the DAWN trial provided the point estimate probability values of mRS scores and 90-day mortality.^18^ Influenced by recent geographical modelling^12^ and our estimates in change in travel time (mode change in travel time in rural England), our base-case analysis assumed a 60 min time to arterial puncture reduction for HEMS (to 300 min) compared with GEMS (360 min). We also account for the number of patients eligible, probabilities of receiving MT backed by advanced imaging and not receiving MT because of spontaneous recanalisation based on previous research by this group.^2^ The annual mortality rate of patients who had stroke is 2.3 times^19^ than that of the age-adjusted, UK population without stroke.^20^ We further differentiate by independent and dependent health state.

For complete cost details, see online supplemental appendix. Briefly, we adopted an NHS perspective and reported 2017–2018 prices using a Bank of England inflation calculator. A microcosting study from five UK neuroscience centres provided the 24-hour procedural costs of MT (Balami, personal communication). The cost in each mRS health state was derived from a previous UK study^3^ with prices inflated to 2018 values. Department of Health national schedule of reference costs (GEMS) and a survey of HEMS providers including Great North Air Ambulance service provided figures on transportation costs. The discount rate (3.5% per annum) accounted for future outcomes being valued less than present outcomes was applied. For specific rural hospitals that this model could apply to, ground-based travel time was calculated by Google maps and reduction in travel time by HEMS was assumed for illustration and discussion.

10.1136/emermed-2019-209039.supp1 Supplementary data

### Outcome measure

Effectiveness was measured in quality-adjusted life Years (QALYs) gained from provision of MT using either transportation strategy. In the model, utility values were assigned to mRS outcome states of patients who had stroke^21^ and the QALY outcome of an individual is the time spent in each state multiplied by the utility of each state.

### Patient and public involvement

DB is a patient representative, part if the research team. DB was involved in all stages of the research process.

### Data analysis strategy

In the base-case analysis on early presenters, deterministic analysis (point estimates) of parameter values are used in the cost-effectiveness (utility) decision model. Probabilistic sensitivity analysis to characterise the uncertainty around the parameter values was conducted. All of the model parameters were jointly varied through credible ranges of values. Using Monte Carlo simulation, parameter values are randomly drawn from the assigned probability distributions and run 1000 times. A cost-effectiveness acceptability curve was produced representing the probability that HEMS transfer will be cost-effective compared with GEMS transfer at a range of willingness-to-pay thresholds. We investigated alternative scenario analyses, such as alternative time-to-treatment intervals and reduction in travel time by 15 min increments. One-way sensitivity analysis of eligibility for MT explored the impact of alternate time horizons (eg, 1–20 years). A secondary analysis of late presenters was also conducted.

Typically, the National Institute for Health and Care Excellence (NICE) recommends an intervention such as a service, technology or drug for use in the NHS if the incremental cost-effectiveness ratio (ICER)—a measure of the additional cost per additional unit of health gained by a proposed intervention over current standard of care—is in the range of £20 000 to £30 000 per QALY gained. Total costs and QALYs were modelled for both HEMS and GEMS to estimate the incremental cost per QALY gained. CHEERS checklist of reporting of economic evaluation was used. All analyses were carried out in TreeAge Pro Healthcare 2020 R1.1 (Morrisontown, New Jersey, USA). This was a modelling study using secondary data sources without personal identifiable data and deemed not to require ethical review.

## Results

We identified 10 small and remote hospitals in England that serve a combined population of approximately 2 million (see online supplemental appendix tables S1, S2). We estimated that up to a maximum of 501 early-presenting patients who had stroke per annum would clinically benefit from MT after secondary transfer using HEMS compared with GEMS (see online supplemental appendix table S3).

### Model results

We based our analysis on the assumption that the mean age of patients was 75 years. To reflect heterogeneity of patients who had stroke, we assumed that age was normally distributed (age range: 60 to 92 years). Baseline estimates of decision tree parameters and the range for all probabilistic sensitivity analyses are provided in online supplemental appendix table S4. Similarly, estimates of Markov model parameters and the range for all probabilistic sensitivity analyses are shown in online supplemental appendix table S5.

**Figure 1:**
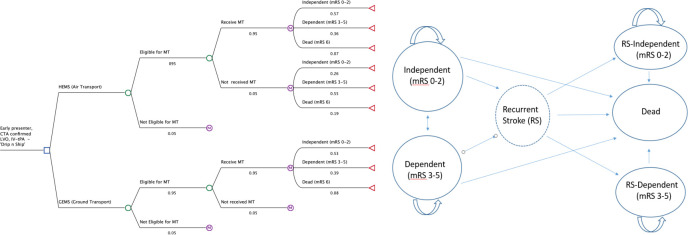
Simplified structure of lifetime economic model with decision tree of transport options along with base-case values for first 3 months (L) and lifetime Markov model of health states. CTA, CT angiography; GEMS, ground emergency medical services; HEMS, helicopter emergency medical services; IV-tPA, intraveneous thrombolysis; LVO, large vessel occulsion; mRS, modified Rankin Scale; MT, mechanical thrombectomy.

In our base-case analysis, for a reduction in the time to reperfusion by 60 min in an early-presenting patient (360 to 300 min), secondary transfer by HEMS for MT was associated with a higher probability of living independently at 90 days than GEMS (0.57 vs 0.53, respectively)—see figure 1. The base-case deterministic and probabilistic results are presented in table 1. In decision analysis, the results of the probabilistic sensitivity analyses are more authoritative. Over a lifetime horizon, using HEMS as a secondary transportation strategy that enables patients to receive MT 60 min earlier results in greater QALYs gained (0.14) but is more costly (£3785). The ICER is £28 027 per QALY gained. The incremental net monetary benefit of the HEMS strategy is £266 for a threshold of £30 000 per QALY gained and favours the GEMS strategy for the lower bound of the NICE threshold (see online supplemental appendix figure S1 for the cost-effectiveness plane).

**Table 1:** Mean lifetime cost-effectiveness analysis (base-case analysis of 1000 early presenter patients aged 75 years)

	HEMS		GEMS		Incremental cost (95% CI)	Incremental QALYs gained (95% CI)	Incremental cost/QALY gained (ICER)	Incremental NMB @20K/QALY	Incremental NMB @30K/QALY
	Mean cost (SE)	Mean QALYs gained (SE)	Mean cost (SE)	Mean QALYs gained (SE)					
Deterministic analysis	£60 132 (£7)	4.37 (0.00)	£56 328 (£6)	4.24 (0.00)	£3804 (£3800 to £3810)	0.14 (0.14 to 0.14)	£27 850	−£1073	+£294
Probabilistic analysis	£60 743 (£319)	4.40 (0.03)	£56 959 (£308)	4.27 (0.03)	£3785 (£3700 to £3870)	0.14 (0.13 to 0.15)	£28 027	−£1084	+£266

Incremental NMB is calculated by first assuming a WTP (£20 000 or £30 000/QALY gained), then converting health benefits (QALYs) into the common metric of GBP. The cost associated with each transport strategy is then subtracted, resulting in the net benefit of each strategy expressed in the monetary units. The difference between the two is the incremental NMB, negative values favour GEMS and positive values favour HEMS. NMB = (E * WTP) – C.

Costs are rounded to nearest whole number. QALYs are rounded to second decimal place.

C, cost; E, effectiveness; GBP, Pound sterling; GEMS, Ground Emergency Medical Services; GEMS, ground emergency medical services; HEMS, helicopter emergency medical services; ICER, incremental cost-effectiveness ratio; NMB, net monetary benefit; QALYs, quality-adjusted life years; WTP, willingness-to-pay threshold.

**Figure 2:**
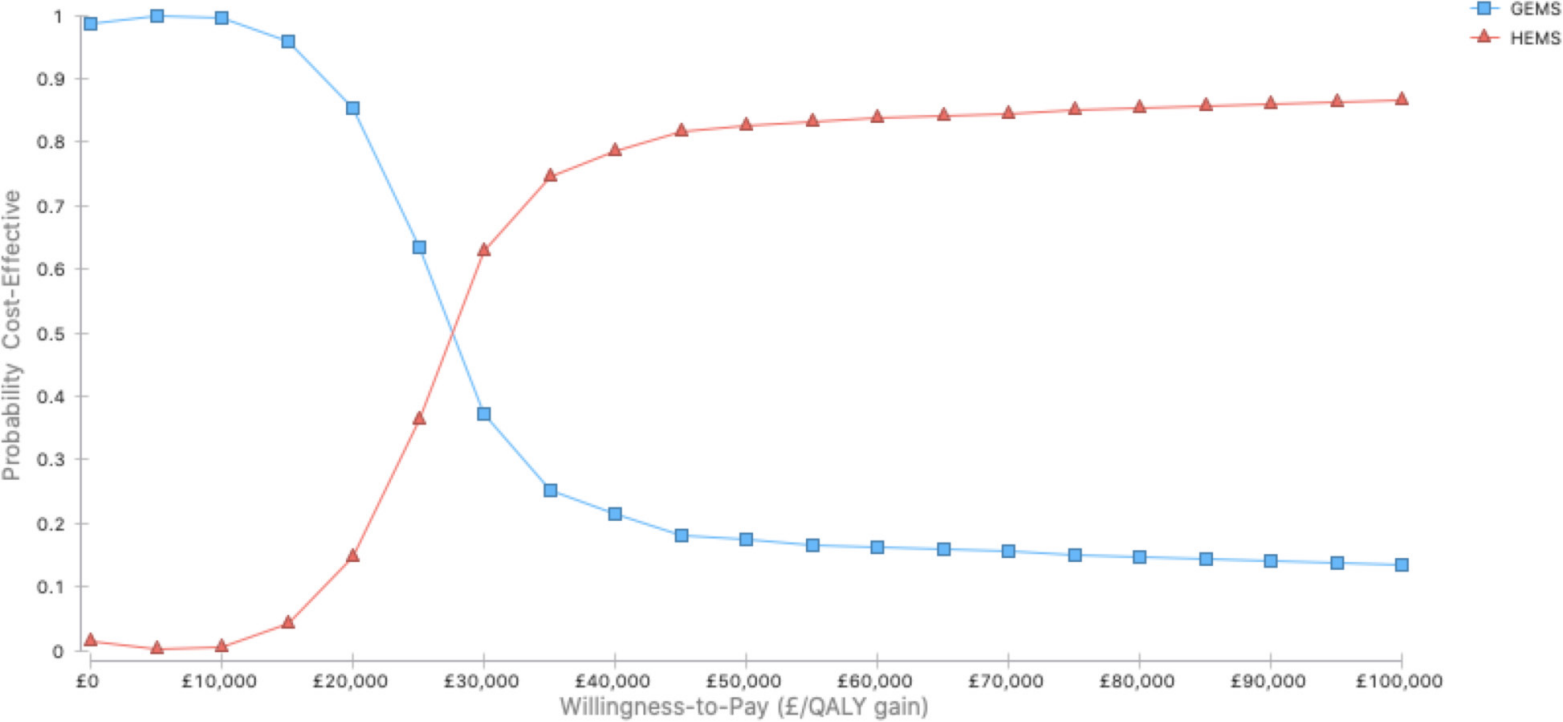
Cost-effectiveness acceptability curve for early presenters by transportation strategy. GEMS, ground emergency medical services; HEMS, helicopter emergency medical services; QALY, quality-adjusted life years.

The cost-effectiveness acceptability curve (figure 2) shows the probability of HEMS being cost-effective for different levels of willingness-to-pay thresholds, compared with GEMS. At £30 000 per QALY threshold, the HEMS strategy had 62.9% probability of being cost-effective.

### Sensitivity and scenario analyses

Using the base-case values, the benefits of the HEMS strategy accrue over time increments with a 5-year time horizon for the benefit of HEMS transportation strategy for early presenters to be achieved (online supplemental appendix table S6). The results are highly sensitive to the proportion of patients in each strategy who are in the dependent state at 90 days, whom have increased annual medical costs to the NHS (first year costs in dependent state £22 706 and £8056 per annum in subsequent years).

When we altered the time of arterial puncture and reductions in travel time, ICERs are higher (see table 2 and online supplemental appendix table S7). This shows that a reduction of at least 60 min is required for the use of HEMS to be considered cost-effective at the upper threshold. The scenario (using base-case values) where those transferred are eligible for MT by HEMS but not by GEMS due to transport time saved, the ICER is £11,679/QALY gained. This assumes that those who travel by GEMS have the same 90-day outcome as those treated with thrombolysis.

**Table 2:** Scenario analysis for early presenters based on travel time difference

HEMS vs GEMS	HEMS (pInd; pDep; pDead)	GEMS (pInd; pDep; pDead)	ICER	% CE at £20k/QALY	% CE at £30k/QALY
Travel time (mins)	60 min difference				
300 vs 360	0.57; 0.36; 0.07	0.53; 0.39; 0.08	£28 027	14.8	62.9
270 vs 330	0.59; 0.35; 0.06	0.53; 0.39; 0.08	£26 318	17.4	70.6
240 vs 300	0.61; 0.33; 0.06	0.57; 0.36; 0.07	£27 415	18.7	64.7
180 vs 240	0.64; 0.30; 0.06	0.61; 0.33; 0.06	£41 370	14.1	26.2
Travel time (min)	45 min difference				
315 vs 360	0.56; 0.37; 0.07	0.53; 0.39; 0.08	£37 545	8.3	25.4
285 vs 330	0.58; 0.35; 0.07	0.55; 0.37; 0.08	£35 266	9.4	27.7
255 vs 300	0.60; 0.34; 0.06	0.57; 0.36; 0.07	£34 864	10.8	29.1
195 vs 240	0.63; 0.31; 0.06	0.61; 0.33; 0.06	£58 414	8.8	16.6

CE, cost-effective; GBA, ground-based ambulance; GEMS, ground emergency medical services; HEMS, helicopter emergency medical services; ICER, incremental cost-effectiveness ratio; QALY, quality-adjusted life year.

Varying the proportion of patients eligible for thrombectomy captures the effect on the ICER if interhospital transfer was futile (table 3). Essentially, the probability that transfer results in thrombectomy occurring is important to the viability of providing the HEMS service for eligible rural patients who had stroke.

**Table 3:** One-way sensitivity analysis of eligibility for MT

Eligibility for MT	HEMS		GEMS		Incremental cost (95% CI)	Incremental QALYs gained (95% CI)	Incremental cost/QALY gained (ICER)	% CE at £20k/QALY	% CE at £30k/QALY
	Mean cost (SE)	Mean QALYs gained (SE)	Mean cost (SE)	Mean QALYs gained (SE)					
0.4	£56 042 (£314)	3.76 (0.03)	£53 284 (£308)	3.75 (0.03)	£2759 (£2710 to £2800)	0.01 (0.01 to 0.02)	£291 127	3.3	10.5
0.5	£56 897 (£314)	3.88 (0.03)	£53 952 (£308)	3.85 (0.03)	£2945 (£2890 to £3000)	0.03 (0.03 to 0.03)	£91 167	4.3	14.6
0.6	£57 752 (£315)	3.99 (0.03)	£54 620 (£307)	3.94 (0.03)	£3132 (£3070 to £3190)	0.06 (0.05 to 0.07)	£56 801	5.1	20.2
0.7	£58 606 (£316)	4.11 (0.03)	£55 288 (£307)	4.03 (0.03)	£3318 (£3250 to £3380)	0.08 (0.07 to 0.09)	£42 562	7.4	27.4
0.8	£59 461 (£317)	4.23 (0.03)	£55 956 (£307)	4.13 (0.03)	£3505 (£3430 to £3580)	0.10 (0.09 to 0.11)	£34 773	9.7	37.5
0.9	£60 316 (£318)	4.34 (0.03)	£56 625 (£307)	4.22 (0.03)	£3691 (£3610 to £3770)	0.12 (0.11 to 0.13)	£29 860	12.9	55.1
1.0	£61 171 (£320)	4.46 (0.03)	£57 293 (£308)	4.31 (0.03)	£3898 (£3790 to £3970)	0.15 (0.14 to 0.16)	£26 479	18.2	68.9

CE, cost-effective; GEMS, Ground Emergency Medical Services; HEMS, Helicopter Emergency Medical Services; ICER, incremental cost-effectiveness ratio; MT, mechanical thrombectomy; QALY, quality-adjusted life year.

The linear assumption of time-to-reperfusion and 90-day mRS score for early presenters cannot be applied to late presenters. Instead, for late presenters we used results from the more recent DAWN trial^18^ which suggested that clinical benefits of MT could occur up to 24 hours from onset of stroke. The probability of 90-day independence is 0.38, 0.48 for dependence and 0.14 for death.^22^ We assumed that the probability of independence increased by two percentage points and dependence decreased by two percentage points if transferred by HEMS. However, this is a very much smaller number of patients than early presenter category comprising only approximately 12% of the total number eligible for MT.^2^ Unsurprisingly, given that there is not the same relationship between time of onset and outcome in late presenters with a favourable imaging profile (and thus eligible for MT), model results indicated that HEMS is dominated by GEMS for late presenters (online supplemental appendix table S9). For late presenters, HEMS is not cost-effective at any acceptable cost-effectiveness threshold to NHS commissioners (online supplemental appendix figures S2,3).

## Discussion

Using the most relevant data, we show the range of cost-effectiveness metrics for consideration of secondary transfer by HEMS from small and remote English hospitals to MT-enabled neuroscience centres, available to NHS commissioners. In our base-case analysis, we consider a reduction of 60 min travel time with an ICER of £28 027. Using the standard NICE cost-effectiveness threshold (£20–£30 000 per QALY), this would suggest that at the lower bound that the service would not be cost-effective but would be within the upper bound. Our sensitivity analysis showed that at least 60 min reduction in travel time is the minimum necessary for HEMS transportation to represent value-for-money at NICE’s upper bound of willingness-to-pay thresholds (£30 000 per QALY). In the scenario of late presenters, using land transport for secondary transfer dominated. In lieu of real-world evidence or a randomised controlled trial, these analyses may be informative to decision-making regarding the optimal service configuration for MT in England, while addressing the critical issue of equity of access in smaller populations more remote from existing (and indeed any future) MT-enabled neuroscience centres.

Empirical international studies show a mixed picture of using HEMS for secondary transfer of patients who had stroke.^6–9 23^ In our modelling study, we only consider the drip-and-ship paradigm. In Alberta, Canada, a Health Technology Optimisation Analysis, incorporating cost-effectiveness analysis, reported the ‘optimal strategy’ as a combination of paradigms (ie, *mothership—direct transfer to MT centre,* drip-and-ship), transport modes (ie, by ground, by flight) and treatment metric options (ie, minimum time to thrombolysis, minimum time to thrombectomy) from a clinical outcomes and health system perspective.^9^ Research incorporating such factors in England would be advantageous; however, modelling this is far more complex as there are 24 existing MT-enabled neuroscience centres in England compared with only two in Alberta. Based on latest SSNAP data, currently ~1200 MT procedures were delivered in 2018/2019 throughout the UK with the overwhelming majority in England.^10^ Out of the estimated 8000 patients eligible for MT in England, cost-effectiveness of HEMS provision pertains to a relatively small number of patients (maximum n≃500). The optimal configuration will always involve a trade-off between minimising time to treatment and sustaining local service provision.^12^

Our modelling work is predicated by the assumption that air transfer will reduce transport time. We then link these hypothetical time savings by HEMS to cost-effectiveness based on expected 90-day outcome. However, delays occur using either mode of transport.^8^ Again, empirical evidence of using HEMS in the NHS service would be beneficial for commissioners to make informed decisions about the potential benefit of secondary transfers for MT. The absence of underpinning HEMS efficiency evidence is a limitation of our modelling work. Our research premise was based on the NHS definition of remote hospitals. However, there are additional English hospitals which do not fully meet the NHS England definition of ‘small and remote’ but are 60–90 min away from a neuroscience centre via GEMS where these findings might also apply, particularly if circumstances and population size preclude the development of a viable local MT centre. Alternatively, the concept of *bringing the stroke treatment to the patient* referred to as the *drip-and-drive* model piloted in Germany involves the neurointerventionist being driven by road to the local hospital.^24^ However, this requires substantial capital (angiographic equipment) and supporting staff investment in the local hospitals plus appreciably larger neurointerventional teams than either a drip-and-ship or mothership model^24^ and is not currently realistic in England.

Reducing futile secondary transfer is imperative. Our base-case model employed a strict eligibility criterion based on advanced imaging in rural hospitals. Our sensitivity analysis showed that 90% of patients need to receive thrombectomy for the HEMS transportation strategy to be considered cost-effective. In reality, even among patients initially deemed good candidates for EVT at the rural hospital, the possibility of futile transfer, either due to worsening infarct or opening of the vessel by alteplase, exists. Also, not all patients with potential thrombectomy will be eligible for flight because of individual characteristics. However, concerns around the potential detrimental impact of HEMS on a patient’s clinical status have not been substantiated.^6^ Nevertheless, HEMS has some intrinsic limitations due to operational aeronautical factors.^5^ HEMS is more vulnerable to weather conditions, aircraft availability and have more restrictive operational hours (10 to 16 hours a day in good weather) than GEMS. An independent report to the Department of Health from 2003 highlighted that unavailability in English services due to weather was approximately 2% of normal flying time.^25^ Another factor that NHS commissioners will need to consider is the governance of air ambulance, as many are charities and not all are under direct NHS deployment.

For the 10 small and remote hospitals across England that this analysis applies to, we assumed time savings by the predominantly charity-funded HEMS providers. Based on the practical and logistical considerations outlined, we feel that contracting on a fee-for-service basis, rather than capital investment, would likely be more appealing to NHS commissioners concerned about budget impact and affordability.

## Conclusion

Helicopter transportation, especially for rural patients, may have a role to play in the national service configuration of stroke services in England. This role should build on the current system to improve effectiveness and outcomes for patients who had stroke irrespective of treatment they receive.
