# Supplementary material for: Secondary transfer of emergency stroke patients eligible for mechanical thrombectomy by air in rural England: economic evaluation and considerations
Source: Emerg Med J. 2020 Nov 10;38(1):33–9. doi: 10.1136/emermed-2019-209039 (PMC7788185; doi:10.1136/emermed-2019-209039)
Supplement: Supplementary data [file emermed-2019-209039supp001.pdf]

Supplemental Appendix

Table S1: Rural NHS hospitals and their populations

Table S2: Remote hospitals, nearest neuroscience centre, travel distance and time, air ambulance service

Table S3: Estimated number of stroke patients and potential costs and health effects implications for air ambulance in rural England

Table S4: Model parameters for decision-tree and range of values for sensitivity analysis

Table S5: Model parameters for Markov model and range of values for sensitivity analysis

Table S6: Time horizons probabilistic sensitivity analysis for early presenters

Table S7: Scenario analysis for early presenters

Table S8a: Scenario analysis for late presenters based on DAWN trial eligibility

Table S8b: Parameters' point estimate for late presenters

Figure S1: Monte Carlo simulations of incremental cost per QALY gained from mechanical thrombectomy within a cohort of 1,000 patients using a Willingness-To-Pay (WTP) threshold of A) £ 20,000 per QALY gained and B) £30,000 per QALY gained for early-presenters.

Figure S2: Monte Carlo simulations of incremental cost per QALY gained from mechanical thrombectomy within a cohort of 1,000 patients for late presenters

Figure S3: Cost-effectiveness acceptability curve for late presenters by transportation strategy

Table S1: Rural NHS hospitals and their populations

Hospital	Location	Total population	Proportion Remote	Remote Population
St Mary's Hospital	Isle of Wight	138,393	1	138,393
North Devon District Hospital	Barnstaple, North Devon	169,852	0.810	137,580
Furness General Hospital	Barrow, Cumbria	111,207	0.617	68,615
Pilgrim Hospital	Boston, Lincolnshire	190,677	0.268	51,101
Hereford County Hospital	Hereford, Herefordshire	182,303	0.236	43,024
Cumberland Infirmary	Carlisle, Cumbria	178,338	0.213	37,986
Scarborough General Hospital	Scarborough, North Yorkshire	194,103	0.105	20,381
Dorset County Hospital	Dorchester, Dorset	162,271	0.042	6,815
Royal Shrewsbury Hospital	Shrewsbury, Shropshire	199,154	0.024	4,780
* Royal Cornwall Hospital	Truro, Cornwall	420,000	0.01	4,200
		**1,946,298**		**512,875**

*Includes Isle of Scilly

Data source: Smyth C, Lorrimer S and Chaplin M. (2016)

Table S2: Remote hospitals, nearest neuroscience centre, travel distance and time, air ambulance service

Hospital	Location	Nearest neuroscience centre	Distance (miles)	Ground Emergency Medical Services Travel Time (Mins)	Helicopter Emergency Medical Service
St Mary's Hospital	Isle of Wright	Southampton	17.0	128	Hampshire & Isle of Wight
North Devon District Hospital	Barnstaple, Devon	Plymouth	59.6	113	Devon
Furness General Hospital	Barrow, Cumbria	Preston	134.0	155	North West Air Ambulance
Pilgrim Hospital	Boston, Lincolnshire	Nottingham	60.3	107	Lincolnshire & Nottinghamshire
Hereford County Hospital	Hereford, Herefordshire	Birmingham	57.8	95	Midlands
Cumberland Infirmary	Carlisle, Cumbria	Newcastle-upon-Tyne	59.7	80	Great North Air Ambulance
Scarborough General Hospital	Scarborough, Yorkshire	Hull	45.8	102	Yorkshire
Dorset County Hospital	Dorchester, Dorset	Southampton	57.3	83	Dorset & Somerset
Royal Shrewsbury Hospital	Shrewsbury, Shropshire	Stoke-on-Trent	41.5	71	Midlands
Royal Cornwall Hospital	Truro, Cornwall	Plymouth	54.7	99	Cornwall

Data Source: Ambulance travel distances and times calculated from Google Maps. This approach was deemed reasonable by previous research by team members that suggested that ambulances in urban areas were able to exceed general traffic speed, whereas, the opposite effect was seen in suburban and rural road networks (McMeekin *et al.* 2014).

Table S3: Estimated number of stroke patients and potential costs and health effects implications for air ambulance in rural England

Hospital	No. of Stroke Patients	No. of Early Presenters	Hypothetical Change in Travel Time by Air (Mins)	Mean Cost Savings (95% CI)	Mean QALYs (95% CI)
St Mary's Hospital	302	38	-90	-£30,458 (-£244,479, £167,373)	4 (0, 11)
North Devon District Hospital	425	53	-60	-£34,974 (-£247,921, £171,028)	4 (0, 12)
Furness General Hospital	219	29	-60	-£20,862 (-£213,713, £156,117)	3 (0, 8)
Pilgrim Hospital	523	66	-90	-£50,244 (-£314,467, £193,994)	6 (0, 17)
Hereford County Hospital	518	66	-60	-£36,682 (-£252,585, £167,275)	5 (0, 13)
Cumberland Infirmary	410	52	-30	-£24,750 (-£193,220, £129,426)	3 (0, 9)
Scarborough General Hospital	250	32	-60	-£22,544 (-£219,912, £157,472)	3 (0, 10)
Dorset County Hospital	407	52	-45	-£25,145 (-£211,643, £150,287)	3 (0, 11)
Royal Shrewsbury Hospital	64	8	-15	-£27,923 (-£151,294, £84,338)	2 (0, 7)
Royal Cornwall Hospital	828	105	-60	-£61,493 (-£319,867, £201,647)	7 (1, 17)

Coughlan D, *et al. Emerg Med J* 2020; 38:1–7. doi: 10.1136/emermed-2019-209039

Data source: Number of stroke patients per rural hospital from SSNAP data. Estimated number of early presenters eligible for mechanical thrombectomy based on McMeekin *et al.* 2017. The hypothetical change in travel time is based on best possible conditions and a readily available helicopter emergency medical services at rural hospital. This does not take account of a myriad of factors that could affect ability to transport patient such as bad weather, normal working hours and medical consideration such as fear of flying or weight/size. Estimated change in travel time with resulting mean cost savings and QALYs are based on discrete event simulation available from McMeekin *et al.* 2019.

Table S4: Model parameters for decision-tree and range of values for sensitivity analysis

Parameter	Point Estimate	Probability Distribution Function	Source
Decision tree			
Eligibility for MT after advanced imaging	0.95	Beta	McMeekin et al. 2017
Received MT after transfer	0.95	Beta	McMeekin et al. 2017
Cost of HEMS	£2,900	Gamma	Great North Air Ambulance (GNAA)
Cost of GEMS	£252	Gamma	NHS Reference Costs 2017-18 (Code: ASS02)
MT by HEMS:			
mRS 0-2 (IV-tPA + MT)	0.57	Conditional beta	Extrapolated Saver et al. 2016
mRS 3-5 (IV-tPA + MT)	0.36	Conditional beta	Extrapolated Saver et al. 2016
mRS 6 (IV-tPA + MT)	0.08	Conditional beta	Extrapolated Saver et al. 2016
MT by GEMS:			
mRS 0-2 (IV-tPA + MT)	0.53	Conditional beta	Extrapolated Saver et al. 2016
mRS 3-5 (IV-tPA + MT)	0.39	Conditional beta	Extrapolated Saver et al. 2016
mRS 6 (IV-tPA + MT)	0.10	Conditional beta	Extrapolated Saver et al. 2016
IV-tPA only:			
mRS 0-2 (IV-tPA only)	0.26	Conditional beta	Ganesalingam et al. 2015
mRS 3-5 (IV-tPA only)	0.55	Conditional beta	Ganesalingam et al. 2015
mRS 6 (IV-tPA only)	0.19	Conditional beta	Ganesalingam et al. 2015

Table S5: Model parameters for Markov model and range of values for sensitivity analysis

Parameter	Point Estimate	Probability Distribution Function	Source
Year 1			
From independent (mRS 0-2) to:			
mRS 0-2	0.955	Conditional beta	Davis et al. 2012
mRS 3-5	0.024	Conditional beta	Davis et al. 2012
Recurrent stroke	0.013	Conditional beta	Davis et al. 2012
Dead	0.008	Conditional beta	Davis et al. 2012
From dependent (mRS 3-5) to:			
mRS 0-2	0.029	Conditional beta	Davis et al. 2012
mRS 3-5	0.919	Conditional beta	Davis et al. 2012
Recurrent stroke	0.013	Conditional beta	Davis et al. 2012
Dead	0.039	Conditional beta	Davis et al. 2012
After Year 1			
From independent (mRS 0-2) to:			
mRS 0-2	0.979	Conditional beta	Davis et al. 2012
mRS 3-5	0	Conditional beta	Davis et al. 2012
Recurrent stroke	0.013	Conditional beta	Davis et al. 2012
Dead	0.008	Conditional beta	Davis et al. 2012
From dependent (mRS 3-5) to:			
mRS 0-2	0	Conditional beta	Davis et al. 2012
mRS 3-5	0.948	Conditional beta	Davis et al. 2012
Recurrent stroke	0.013	Conditional beta	Davis et al. 2012
Dead	0.039	Conditional beta	Davis et al. 2012
**Recurrent stroke to:**			
(IV-tPA + Throm) mRS 0-2	0.867	Conditional beta	Davis et al. 2012
(IV-tPA + Throm) mRS 3-5	0.104	Conditional beta	Davis et al. 2012
(IV-tPA + Throm) recurrent stroke	0	Conditional beta	Davis et al. 2012
(IV-tPA + Throm) dead	0.029	Conditional beta	Davis et al. 2012
(IV-tPA alone) mRS 0-2	0.834	Conditional beta	Davis et al. 2012
(IV-tPA alone) mRS 3-5	0.137	Conditional beta	Davis et al. 2012
(IV-tPA alone) recurrent stroke	0	Conditional beta	Davis et al. 2012
(IV-tPA alone) dead	0.029	Conditional beta	Davis et al. 2012
**Health Utilities**			
Independent	0.74	Beta	Sandercock et al. 2002
Dependent	0.38	Beta	Sandercock et al. 2002

Recurrent stroke	0.34	Beta	Sandercock et al. 2002
**Costs**			
IV-tPA (Drug & 24-hr care)	£2,339.34	Gamma	BNF 2018 & Davis et al. 2012
Thrombectomy (24-hour)	£8,479.27	Gamma	Balami et al. 2018
**First 3 months:**			
Independent	£7,773.00	Gamma	Ganesalingam et al. 2015
Dependent	£16,632.70	Gamma	Ganesalingam et al. 2015
Fatal	£10,658.07	Gamma	Ganesalingam et al. 2015
**Ongoing per 3 months:**			
Independent	£748.53	Gamma	Youman et al 2003
Dependent	£2,014.12	Gamma	Youman et al 2003
**Recurrent Stroke**			
Average NHS stroke patient	£13,935.53	Gamma	Xu et al.2017
**Mortality Factor**			
Independent	1.16	-	Davis et al. 2012, Ganesalingam et al. 2015
Dependent	5.65	-	Davis et al. 2012, Ganesalingam et al. 2015

Note: NHS costs were valued at 2017-2018 prices using a Bank of England inflation calculator.

Coughlan D, *et al. Emerg Med J* 2020; 38:1–7. doi: 10.1136/emermed-2019-209039

Table S6: Time horizons probabilistic sensitivity analysis for early presenters

Time Horizon	Helicopter Emergency Medical Service		Ground Emergency Medical Services		Incremental Cost (95% CI)	Incremental QALYs gained (95% CI)	Incremental Cost/QALY gained (ICER)	% CE at £20K/QALY	% CE at £30K/QALY
	Mean Cost (SE)	Mean QALYs gained (SE)	Mean Cost (SE)	Mean QALYs gained (SE)					
1-year	£26,765 (£41)	0.64 (0.00)	£24,325 (£41)	0.63 (0.00)	£2,440 (£2,380, £2,500)	0.02 (0.02, 0.02)	£139,306	0.0	0.0
3-year	£35,433 (£53)	1.53 (0.00)	£32,679 (£53)	1.49 (0.00)	£2,743 (£2,680, £2,800)	0.04 (0.04, 0.04)	£65,803	0.1	2.8
5-year	£42,375 (£79)	2.27 (0.01)	£39,374 (£78)	2.21 (0.01)	£3,000 (£2,940, £3,060)	0.06 (0.06, 0.07)	£47,910	1.2	11.1
10-year	£53,580 (£175)	3.51 (0.00)	£50,130 (£170)	3.40 (0.01)	£3,450 (£3,380, £3,520)	0.10 (0.09, 0.11)	£34,066	7.9	34.4
15-year	£58,547 (£256)	4.10 (0.02)	£54,873 (£248)	3.98 (0.02)	£3,674 (£3,590, £3,760)	0.12 (0.11, 0.13)	£29,961	12.2	54.4
20-year	£60,242 (£300)	4.33 (0.03)	£56,484 (£289)	4.20 (0.03)	£3,758 (£3,670, £3,840)	0.13 (0.12, 0.14)	£28,533	14.1	61.3

Table S7: Scenario analysis for early presenters

**Travel Time (Mins)**	**30 minutes difference**				
HEMS vs GEMS	***HEMS (pInd; pDep; pDead)***	***GEMS (pInd; pDep; pDead)***	***ICER***	***% CE at £20k/QALY***	***% CE at £30k/QALY***
330 vs 360	0.55;0.37;0.08	0.53;0.39;0.08	£70,876	5.0	12.8
300 vs 330	0.57;0.36;0.07	0.55;0.37;0.08	£52,939	6.2	15.5
270 vs 300	0.59;0.35;0.06	0.57;0.36;0.07	£52,629	7.1	16.1
210 vs 240	0.62;0.32;0.06	0.61;0.33;0.06	£151,866	6.7	12.4

**Travel Time (Mins)**	**15 minutes difference**				
HEMS vs GEMS	***HEMS (pInd; pDep; pDead)***	***GEMS (pInd; pDep; pDead)***	***ICER***	***% CE at £20k/QALY***	***% CE at £30k/QALY***
345 vs 360	0.54;0.38;0.08	0.53;0.39;0.08	£158,896	2.9	8.4
315 vs 330	0.56;0.37;0.07	0.55;0.37;0.08	£85,862	4.2	10.4
285 vs 300	0.58;0.35;0.07	0.57;0.36;0.07	£155,374	4.8	10.8
225 vs 240	0.62;0.33;0.05	0.61;0.33;0.06	£83,744	6.7	14.0

Coughlan D, *et al. Emerg Med J* 2020; 38:1–7. doi: 10.1136/emermed-2019-209039

Table S8a: Scenario analysis for late presenters based on DAWN trial eligibility

	HEMS		GEMS					
	Mean Cost (SE)	Mean QALYs gained (SE)	Mean Cost (SE)	Mean QALYs gained (SE)	Incremental Cost (95% CI)	Incremental QALYs gained (95% CI)	Incremental Cost /QALY gained (ICER)	Incremental Net Monetary Benefit
**Deterministic Analysis**	£54,632 (£6)	3.41 (0.00)	£51,217 (£5)	3.42 (0.00)	£3,416 (£3,411, £3,421)	-0.01 (-0.01, -0.01)	Dominated	-
**Probabilistic Analysis**	£55,000 (£316)	3.41 (0.03)	£51,536 (£310)	3.42 (0.03)	£3,463 (£3,378, £3,548)	-0.01 (-0.01, -0.01)	Dominated	-

Table 8b: Parameters' point estimate for late presenters

**Parameter**	**Point Estimate**	**Probability Distribution Function**	**Source**
Eligibility for MT after advanced imaging	0.50	Beta	Expert opinion
Received MT after transfer	0.50	Beta	Expert opinion
MT by HEMS:			
mRS 0-2 (IV-tPA + MT)	0.40	Conditional beta	Albers et at. 2018 (DAWN trial)
mRS 3-5 (IV-tPA + MT)	0.46	Conditional beta	Albers et at. 2018 (DAWN trial)
MT by GEMS:			
mRS 0-2 (IV-tPA + MT)	0.38	Conditional beta	Albers et at. 2018 (DAWN trial)
mRS 3-5 (IV-tPA + MT)	0.48	Conditional beta	Albers et at. 2018 (DAWN trial)

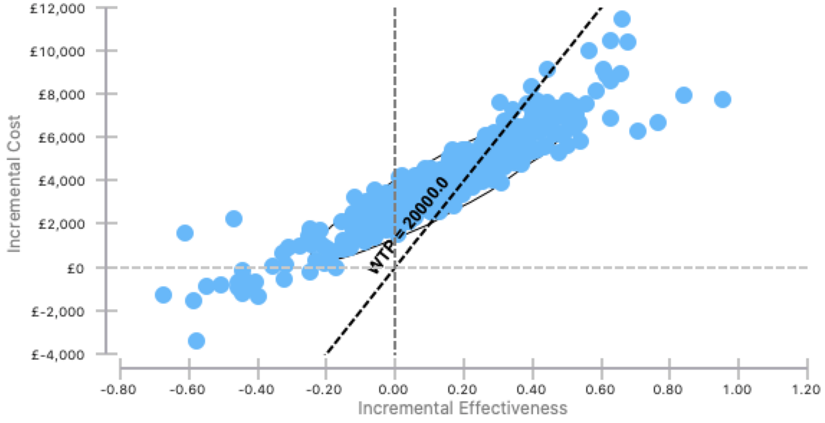

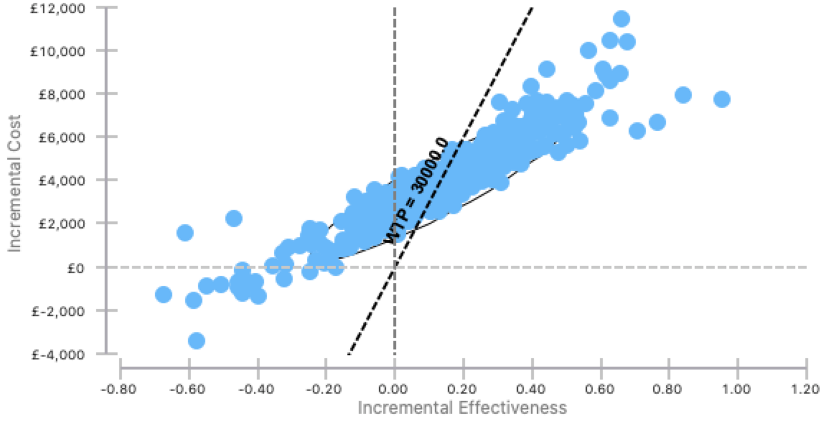

Figure S1: Monte Carlo simulations of incremental cost per QALY gained from mechanical thrombectomy within a cohort of 1,000 patients using a Willingness-To-Pay (WTP) threshold of A) £ 20,000 per QALY gained and B) £30,000 per QALY gained for early-presenters. Also included is the 95% credible region of the ICER using the confidence ellipse method.

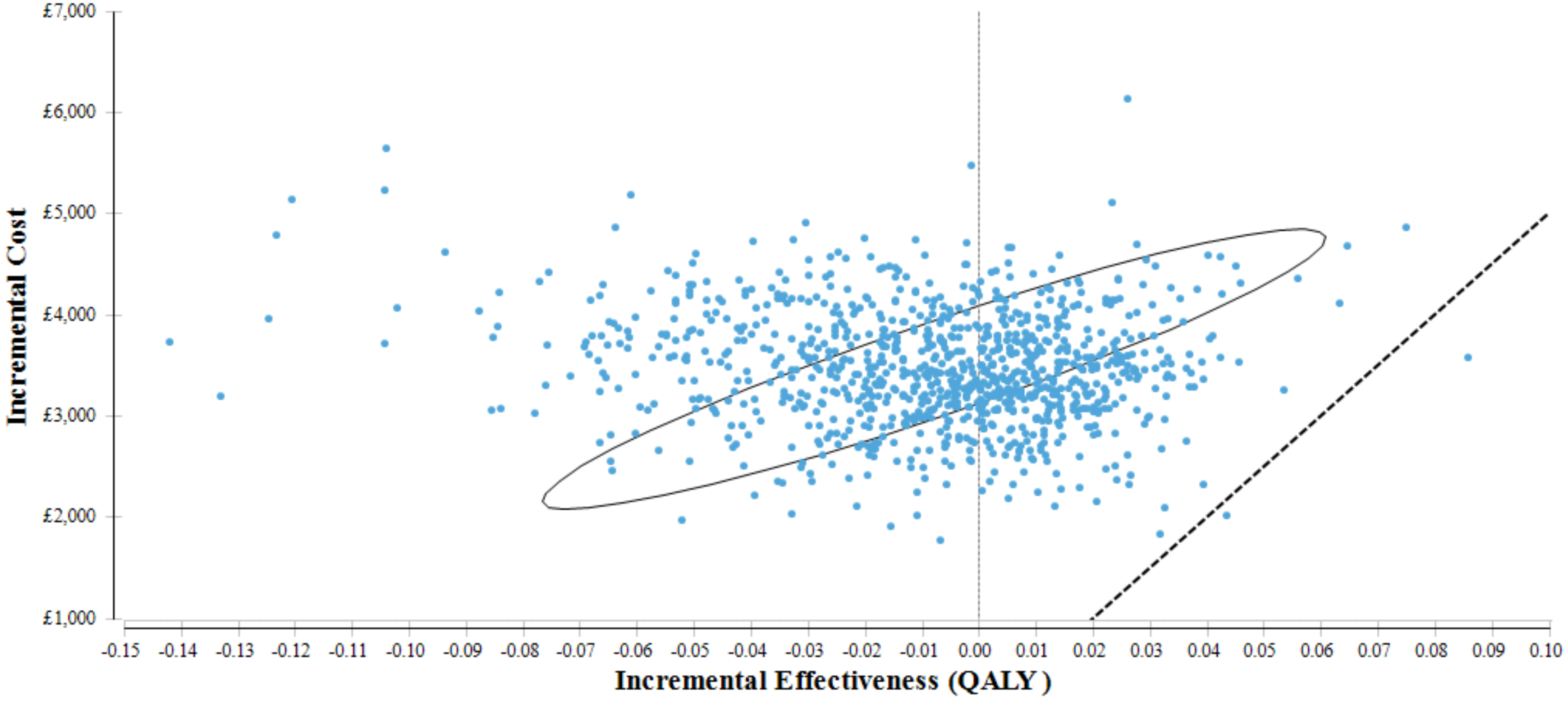

Figure S2: Monte Carlo simulations of incremental cost per QALY gained from mechanical thrombectomy within a cohort of 1,000 patients using a Willingness-To-Pay (WTP) threshold of £50,000 per QALY gained for late-presenters. Also included is the 95% credible region of the ICER using the confidence ellipse method.

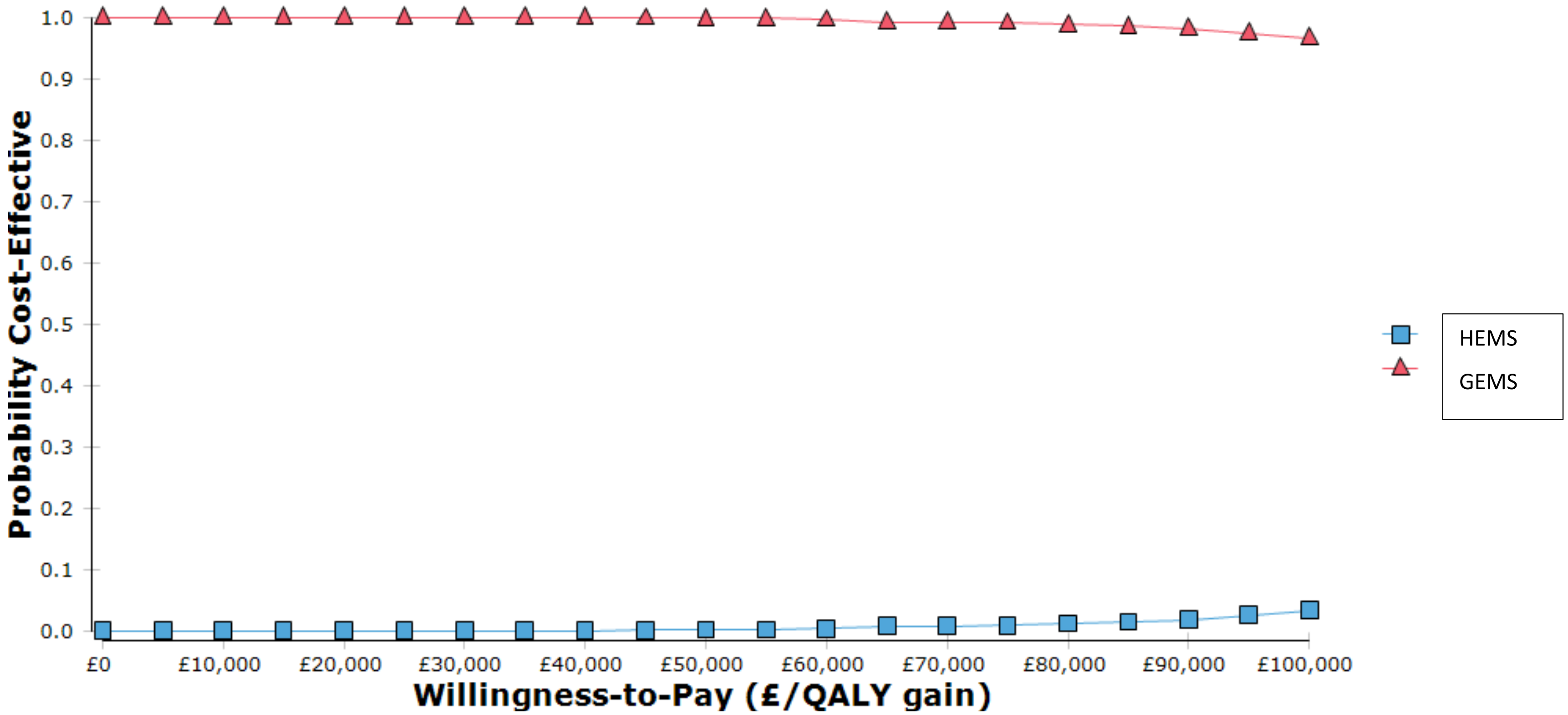

Figure S3: Cost-effectiveness acceptability curve for late presenters by transportation strategy

References:

Alberts MJ, Ollenschleger MD, Nouh A. DAWN of a New Era for Stroke Treatment: Implications of the DAWN Study for Acute Stroke Care and Stroke Systems of Care. *Circulation* 2018: **137**(17):1767-69

Balami JS, McMeekin P, White PM, Flynn D, Wiggam I, Roffe C, Flynn P, Chembala J, Natarajan I, Dixit A, Hunter AM, Foddy L, Hopkins E, Coughlan D, Lumley H, Rice S, Burgess D, Craig D, Buchan AM, Ford GA, Gray A. Case Costing of Mechanical thrombectomy for acute ischaemic stroke in routine clinical setting: Cost differences between mothership vs drip and ship. *Presented at United Kingdom Stroke Forum (UKSF)*. 2018, Telford, UK.

Bank of England Inflation Calculator. https://www.bankofengland.co.uk/monetary-policy/inflation/inflation-calculator [Last accessed: 7th May 2019]

British National Formulary (BNF) 76. September 2018. Available from https://www.bnf.org/products/bnf-online/ [Last accessed: 16th April 2019]

Davis,S., Holmes,M., Simpson,E., Sutton,A. Alteplase for the treatment of acute ischaemic stroke (review of technology appraisal 122): A Single Technology Appraisal. ScHARR, The University of Sheffield, 2012.

Department of Health. National schedule of reference costs, 2017-18. Available from: https://improvement.nhs.uk/resources/reference-costs/ [Last accessed: 16th April 2019]

Ganesalingam J, Pizzo E, Morris S, *et al.* Cost-Utility Analysis of Mechanical Thrombectomy Using Stent Retrievers in Acute Ischemic Stroke. *Stroke* 2015;**46**:2591–8

Great North Air Ambulance. 2019. Darlington. UK – Available from: https://www.greatnorthairambulance.co.uk/

McMeekin P, Gray J, Ford GA, Duckett J, Price CI. A comparison of actual versus predicted emergency ambulance journey times using generic geographic information system software. *Emerg Med J* 2014; **31(9),** 758-762

McMeekin P, White P, James MA, *et al.* Estimating the number of UK stroke patients eligible for endovascular thrombectomy. *Eur Stroke J* 2017; **2**: 319–26

McMeekin P, Flynn D, Allen M, *et al.* Estimating the effectiveness and cost-effectiveness of establishing additional endovascular Thrombectomy stroke Centres in England: a discrete event simulation. *BMC Health Serv Res* **19**, 821 (2019)

Office for National Statistics (ONS). National life tables, UK Statistical bulletins 2018 - Available from: https://www.ons.gov.uk/peoplepopulationandcommunity/birthsdeathsandmarriages/lifeexpectancies/bulletins/nationallifetablesunitedkingdom/2015to2017 [Last accessed: 16th April 2019]

Sandercock P, Berge E, Dennis M, *et al.* A systematic review of the effectiveness, cost-effectiveness and barriers to implementation of thrombolytic and neuroprotective therapy for acute ischaemic stroke in the NHS. *Health Technol Assess* 2002; **6**:1–112.

Saver JL, Goyal M, Lugt A van der, *et al.* Time to Treatment With Endovascular Thrombectomy and Outcomes From Ischemic Stroke: A Meta-analysis. *JAMA* 2016;**316**:1279–89.

Sentinel Stroke National Audit Programme (SSNAP). Cost and Cost-effectiveness analysis – Technical report. 2016. Royal College of Physicians, London. Available from: https://www.strokeaudit.org/SupportFiles/Documents/Health-Economics/Health-economic-report-2016.aspx [Last accessed: 16th April 2019]

SSNAP – Clinical Audit. Royal College of Physicians, London. Available from: https://www.strokeaudit.org/results/Organisational/National-Organisational.aspx

Smyth C, Lorrimer S, Chaplin M. Advisory Committee on Resource Allocation (acra-2015-18A: Unavoidable smallness due to remoteness - identifying remote hospitals. Available at: https://www.england.nhs.uk/wp-content/uploads/2016/04/acra-2015-18A-unavoidable-smallness-upd.pdf (Last accessed: 20th April 2020).

Stroke Association. State of the nation – Stroke statistics February 2018. Available from: https://www.stroke.org.uk/system/files/sotn_2018.pdf (Last accessed: 16th April 2019)

Youman P, Wilson K, Harraf F, Kaira L. The economic burden of stroke in the United Kingdom. *Pharmacoeconomics* 2003: **21(S1)**:43-50

Xu XM, Vestesson E, Paley L, Desikan A, Wonderling D, Hoffman A, Wolfe CD, Rudd AG, Bray BD. The economic burden of stroke care in England, Wales and Northern Ireland: Using a national stroke register to estimate and report patient-level health economic outcomes in stroke. *Eur Stroke J* 2018; 3(1):82-91
